# Protein Z modulates the metastasis of lung adenocarcinoma cells

**DOI:** 10.1515/biol-2022-0667

**Published:** 2023-07-28

**Authors:** Jin Peng, Kai-Ying Yang, Huan Li, Shan-Shan Zheng, Xue-Yi Pan

**Affiliations:** Department of Hematology, The First Affiliated Hospital of Guangdong Pharmaceutical University, 19 Nong Lin Road, Yuexiu District, Guangzhou 510080 Guangdong, China

**Keywords:** protein Z, non-small cell lung cancer cells, metastasis, invasion, epithelial–mesenchymal transition pathway

## Abstract

Protein Z (PZ), a vitamin-K-dependent anticoagulant glycoprotein, is reported to be highly expressed in various malignant tissues and correlated with a poor prognosis in patients with lung cancer. This study aimed to investigate the pathological activity of PZ on lung cancer cell migration, invasion, and metastasis. PZ was assessed by Western blot in three non-small-cell lung cancer cell lines (A549, H1299, and H1975). Meanwhile,western blot was used to detect the expression of EMT pathway-related proteins (Slug, Vimentin, and N-cadherin) in the A549 cells knocked down with siRNA. The cellular proliferation, migration, and invasion were detected by Cell Counting Kit (CCK)-8, wound healing, and Transwell assays in the A549 cells. The results showed that PZ expression was higher in A549, H1299, and H1975 cells, according to Western blot. CCK-8, wound healing, and Transwell assays showed that knockdown of PZ significantly decreased cellular proliferation, migration, and invasion, as well as the protein levels of Slug, Vimentin, and N-cadherin in the A549 cells. In conclusion, the pro-metastasis activity of PZ may modulate the epithelial–mesenchymal transition pathway in lung cancer A549 cells.

## Graphical abstract

Epithelial–mesenchymal transition (EMT)-related proteins (Slug, vimentin, and N-cadherin) are downregulated by Protein Z silencing, which significantly prohibits cellular proliferation and EMT activity (decrease of cellular migration and invasion) in non-small-cell lung cancer cells.

**Figure:**
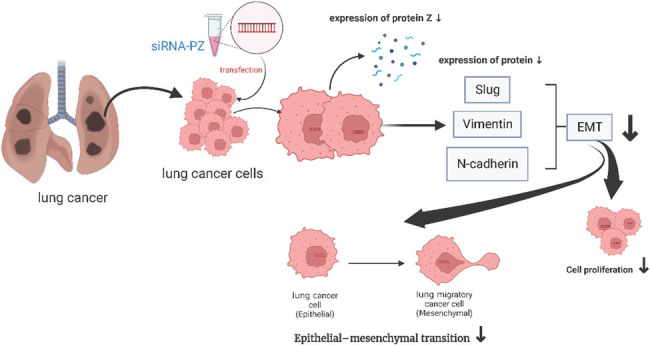

## 1 Introduction

Lung carcinoma is a malignancy characterized by uncontrolled growth in lung tissues and metastasis rapidly to other organs. Lung cancer, also known as primary bronchial lung cancer, has the highest morbidity and mortality rates worldwide. Lung cancer is the leading cause of cancer death in the United States and worldwide [1,2,3,4]. According to histological characteristics, it can be divided into small-cell lung cancer and non-small-cell lung cancer (NSCLC), among which NSCLC accounts for 80–85% of the incidence of lung cancer [5,6]. In recent years, the incidence rate of lung cancer has increased due to the alteration of outside air pollution, unclean living and diet, self-emotional influence, and many other reasons. So far, the therapeutic methods of the NSCLCs mainly adopt molecular targeted therapy and surgery. However, due to the long-term applications of molecular targeted drugs, the patients would become insensitive to the drugs, resulting in drug resistance [6,7]. Understanding the genetic characteristics and biomolecular mechanisms of lung cancer significantly promote the development of effective novel molecular target drugs (such as epidermal growth factor receptor tyrosine kinase inhibitor: Osmertinib) and immunochemical drugs (such as CRLX101 and TAS-102) [8,9,10], which improved prognosis in the patients with lung cancer. However, lung cancer metastasis and invasion continue to confound doctors in clinical practice, particularly in adenocarcinomas, where early metastasis is common. An initial diagnosis of lung cancer patients with advanced metastasis almost means a poor prognosis. Therefore, it is urgent to acquire further detailed mechanisms regarding lung cancer metastasis and invasion to explore new therapeutic targets for lung cancer treatment.

Protein Z (PZ) [11,12] is a serum anticoagulation glycoprotein synthesized in the liver. It can bind to a Z-dependent protease inhibitor (ZPI). PZ acts synergistically with ZPI to inhibit factor Xa to enhance anticoagulation significantly [13]. In cases of PZ deficiency, inhibition of factor Xa through ZPI will be reduced, increasing both coagulant activity and thrombotic risk [14]. Heparin is closely related to PZ. Studies have shown that heparin-activated PZ-dependent protease inhibitors can prohibit the activation of PZ by prolonging the heparin-binding site through activation [15]. PZ has been reported to be overexpressed in many malignancies, including gastric, colon, and lung cancers [16,17,18,19,20,21,22]. It has also been noticed that decreased serum concentration of PZ is closely associated with thalidomide treatment in multiple myeloma patients. Whether PZ is a prognostic risk must be further verified [23]. Our previous study found that the level of PZ is associated with the progression of cancer, suggesting that PZ may be an independent factor for poor prognosis [24]. However, the pathological activity of PZ during lung cancer invasion and metastasis is still uncovered.

The epithelial–mesenchymal transition (EMT) is one of the main pathways in cancer metastasis. The occurrence and development of lung cancer is closely related to the theory of epithelial interstitial transformation [25,26,27]. EMT is a pathological process in tumor cells. Epithelial cells can acquire mesenchymal phenotypes under stimulation by tumor microenvironmental factors, such as epidermal growth factor, endothelin 1, and bone morphogenetic protein [28], leading to metastasis and invasion of tumor cells. It has also been shown that specific transcription factors (TFs), such as SNAIL/SNAI1, SLUG/SNAI2, TWIST, and ZEB family TFs, downregulate E-cadherin and upregulate N-cadherin and Vimentin proteins during EMT [29]. Slug, Vimentin, and N-cadherin proteins are essential during the EMT process. The Slug protein has been reported to upregulate cancer metastasis in various tumors. Vimentin has been found in pancreatic, breast, colon, and ovarian cancers. It closely relates to cancer's clinical stage and prognosis [30,31]. Slug is revealed to cooperate with Vimentin in the progression of breast cancer metastasis [32]. Slug protein is highly expressed in the histogenesis of pleomorphic adenoma (PA) and is an essential regulatory factor of EMT in PA [33]. Co-expression of Vimentin and Slug proteins induces a worse prognosis in neoplasms [34]. N-cadherin is expressed in multiple forms of cancers and plays a significant role in the EMT pathway [35,36,37,38,39]. EMT activation can decrease the clinical efficacy of biomolecular targeting therapy in lung adenocarcinoma, A549 cells, and other lung cancer cell lines [40,41]. It is hypothesized that PZ could influence lung cancer metastasis and invasion through the EMT pathway.

In this study, behaviors of the PZ on cellular migration, invasion, and metastasis were investigated through siRNA knockdown in lung cancer cell lines. Our observations demonstrated that PZ significantly influences lung cancer invasion. This pro-metastasis activity of PZ may be achieved by modulation of the EMT pathway.

## 2 Methods

### 2.1 Materials

Human lung adenocarcinoma cell lines A549 was purchased from the Shanghai Science Research Cell Resource Center, and H1299 and H1975 were supplied by Shanxi Rosetta stone biotechnology Ltd. The Cell Counting Kit (CCK)-8 was purchased from the Dalian Meilun Biology Science and Technology (Dalian, China). Tanswells were purchased from Corning (Corning, NY, USA). Mouse polyclonal anti-β-actin antibody was obtained from Boaosen Biotechnology (Beijing, China). Mouse monoclonal anti-PZ antibody was purchased from Boaosen Biotechnology (Beijing, China). Rabbit monoclonal anti-Slug (9585T) and anti-Vimentin antibodies (5741T) were purchased from Cell Signaling Technology (Danvers, MA, USA). Rabbit monoclonal anti-N-cadherin antibody was purchased from Bosterbio (Wuhan, China).

### 2.2 Cell culture

NSCLC cell lines, A549, H1299, and H1975 cells, were cultured in RPMI-1640 (Gibco Laboratories, USA) supplemented with 10% fetal bovine serum (FBS; Gibco Laboratories, USA). All cell lines were incubated at 37°C in 5% CO_2_ with the media that was exchanged every 24 h or 48 h according to the growth condition of the cells.

### 2.3 Western blot assay

A549, H1299, or H1975 cells were collected and washed with phosphate-buffered saline (PBS) three times, lysed with standard RIPA buffer (Beyotime, China) on ice for 30 mins, and centrifuged at 13,000×*g* for 10 mins at 4°C. The supernatants were collected as detecting samples. The protein concentration was determined using a BCA kit (Thermo Fisher Scientific, Waltham, MA, USA) according to the manufacturer’s instructions. Aliquot proteins of the samples were loaded on a 10% sodium dodecyl sulfate–polyacrylamide gel electrophoresis, separated by electrophoresis, and transferred onto a poly(vinylidene fluoride) membrane (Millipore, Bedford, MA, USA). After reaction with the corresponding primary and secondary antibodies, the blotted membranes were visualized using enhanced chemiluminescence (Meilun, Dalian, China) reagents.

### 2.4 Transfection of siRNAs

A549 lung adenocarcinoma cells (1.5 × 10^5^ cells/well) were seeded into six-well plates. Following a 24 h incubation, the cells at 70–80% confluence were transfected with the corresponding PZ-siRNAs (Ribobio, Guangzhou, China) (with a final concentration of 100 nM) in serum-free media using riboFECT CP Transfection Kit according to the instructions provided in the siRNA kit (Ribobio, Guangzhou, China). The sequences of the PZ-siRNA are shown in Table 1. The negative control cells were transfected with noncoding siRNA-NC (Ribobio, Guangzhou, China). The blank control cells were treated with the transfection solution without siRNA as described earlier (mock transfection). The transfected cells were harvested at the indicated time points for Western blot analysis following the aforementioned procedures.

**Table 1:** siRNA sequences

siRNA-PZ001: TCACCAAGGTCTCCAGGTA
siRNA-PZ002: CTGTGGTGGTGTTATAATA
siRNA-PZ003: GGTTTAAACAGATCATGAA

### 2.5 Wound healing assay

Cellular migration was detected by wound healing assay. Briefly, A549 cells were transfected with siRNA-PZ or siRNA-NC on a glass slide in a six-well plate and cultured for 48 h. The cells were then scraped with a fine end of 1 mL pipette tip and washed thrice with PBS to remove detached cells. Images were captured to record the original position (time 0) of the cells. The cells were incubated in growth media with 1% FBS for 24 h and 48 h and pictured at 24 h or 48 h. The migration rate was estimated by dividing the migrated area by the total space area. The migrated area was calculated by subtracting the area of time 0 from that of time 24 h or 48 h in the wounded space.

### 2.6 Transwell assays

Cellular migration and invasion assays were performed using Transwells with a polycarbonate nucleopore membrane (8 μm pore size; Corning, USA). For the haptotactic migration assay, the bottom chamber was filled with media containing 20% FBS. A549 cells (5 × 10^4^) suspended in serum-free media were seeded in the top chamber and allowed to migrate for 12 h. The bottom chambers were again filled with media containing 20% FBS for invasion assay. However, the top chamber of the Transwell was coated with Matrigel (BD Biosciences, San Jose, CA, USA) diluted in media without FBS (1:8). The cells (1 × 10^5^ cells in serum-free media) were seeded in the top chamber on the Matrigel and allowed to invade overnight or for 24 h. Following, either the migrated or the invasive cells that had passed through the membrane to the bottom membrane were fixed, stained with crystal violet, and counted in five independent microscopic fields at 20× magnification or all cells/filter.

### 2.7 Cell proliferation assay

A549 cells were transfected in six-well plates with siRNA-PZ or siRNA-NC. Forty-eight hours later, the cells were detached, diluted, seeded into a 96-well plate (5 × 10^3^ cells/well), and incubated overnight. After CCK-8 dye (10 μl; Meilun, Dalian, China) was added to each well, the initial absorbance (optical density (OD)) of each well was measured. Cells were further incubated for 24 h and 48 h, and the OD was measured at each time point.

## 3 Results

### 3.1 Higher PZ expression in A549 and H1975 cells

**Figure 1:**
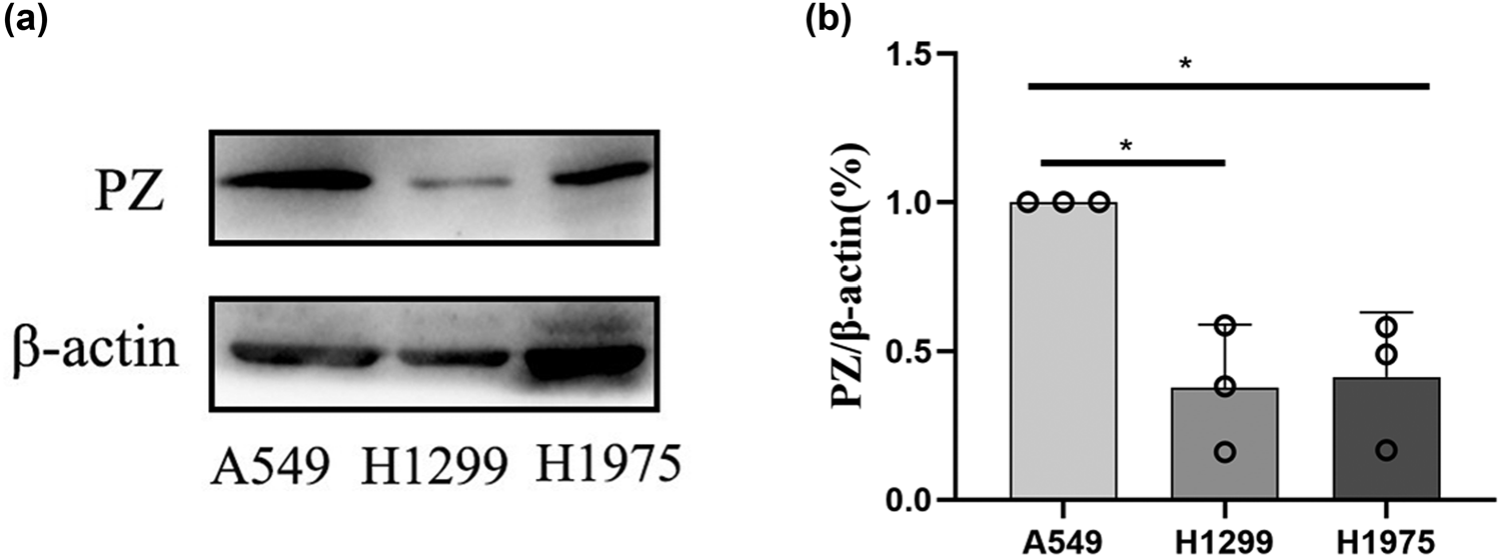
PZ expression in A549, H1299, and H1975 cells. (a) Representative images of Western blotting showed the expression levels of PZ in A549, H1299, and H1975 cells. β-actin was used as an internal standard. (b) The average expression levels of the PZ protein were quantified from three independent assays. * indicates the significant difference between the groups (*p* < 0.05).

PZ expression was detected in A549, H1299, and H1975 NSCLC cell lines by Western blot. The results indicated that the PZ protein expression level was significantly higher in A549 cells compared to either H1299 or H1975 cells (*p* < 0.05; Figure 1a). The PZ protein level was significantly lower in H1299 cells compared to A549 and H1975 cells (*p* < 0.05; Figure 1b).

### 3.2 Knockdown of PZ inhibited the PZ expression in A549 cells

**Figure 2:**
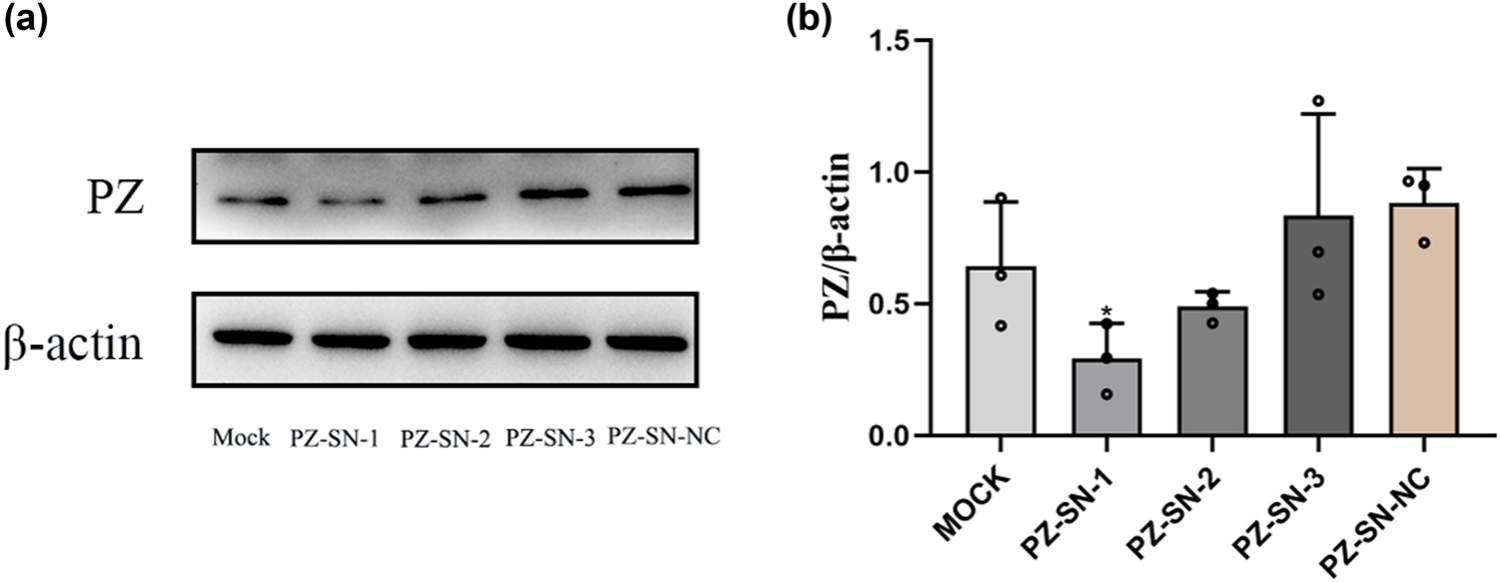
Knocking-down efficiency of the PZ-siRNAs in A549 cells. (a) The levels of PZ in A549 cells transfected with indicated PZ-siRNAs (siPZ1-siPZ3) were detected by Western blot. The mock sample was treated with transfection reagent alone; the si-NC sample was transfected with noncoding siRNA (scramble control). (b) Relative levels of the PZ in the corresponding group are quantified by densitometry analysis from three independent assays. * indicates the significant difference between the groups (*p* < 0.05).

To address the pathological activity of PZ, the PZ protein expression was analyzed in the A549 cells transfected with either PZ-siRNAs or PZ-SN-NC. The results indicated that the expression levels of PZ were significantly decreased after transfection with either PZ-siRNA-001, PZ-siRNA-002, or PZ-siRNA-003 in A549 cells compared to the mock control cells and negative control cells (transfected with other scramble siRNAs) (*p* < 0.05; Figure 2). Among the PZ-siRNAs, the PZ-siRNA-001 showed the maximal inhibitive effect in A549 cells. Therefore, the PZ-siRNA-001 was applied in the subsequent experiments.

### 3.3 PZ knocking-down significantly decreased the cellular migration and invasion in the A549 cells

**Figure 3:**
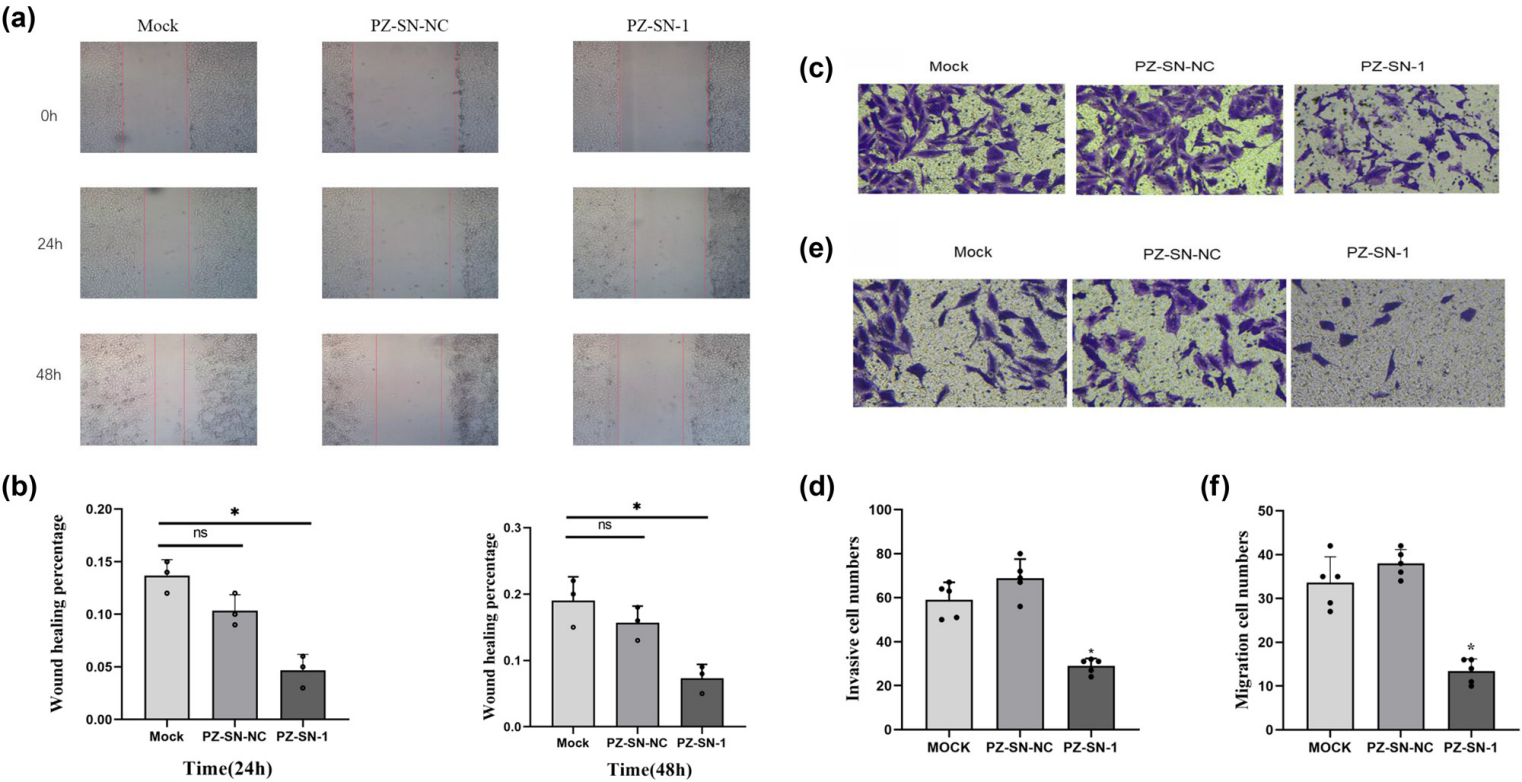
Impact of PZ knocking-down on cellular migration and invasion in A549 cells. (a) Cellular migration is detected in wound healing assay in the mock (left column), PZ-siRNA-01 (right column), and siRNA-NC (middle column) A549 cells for 24 h (middle panels) and 48 h (lower panels). 0 h represents the begging time of the assays. (b) The average cellular migration rates of the corresponding samples in A are estimated from three independent assays. (c) Cellular invasion assay is conducted with the Transwell method in the mock (left), PZ-siRNA-1 (right, PZ-SN-1), and siRNA-NC (middle, PZ-SN-NC) A549 cells. (d) Average cellular invasion rates of the corresponding samples in C are calculated from three independent assays. (e) Cellular migration assay is performed in the indicated samples. (f) Average cellular migration rates of the samples in E are analyzed from three independent assays. * indicates the significant difference between the groups (*p* < 0.05).

To assay the metastatic effect of the PZ, the cellular migration and invasion were analyzed in the A549 cells transfected with either PZ-siRNA-001 or PZ-SN-NC. The wound healing assay results indicated that the cellular migration was significantly reduced in A549 cells transfected with PZ-siRNA-001 compared to the mock-transfected cells and the siRNA-NC-transfected cells at both 24 h and 48 h (*p* < 0.05; Figure 3a and b). These results were verified again by the Transwell method (Figures 3e and f; *p* < 0.05).

The invasion assay results showed that the number of invasive A549 cells was significantly reduced when the cells were transfected with PZ-siRNA-001 compared to those of the mock-transfection or siRNA-NC transfected cells (*p* < 0.05; Figure 3c and d).

### 3.4 PZ-knockdown decreased the cellular proliferation in the A549 cells

**Figure 4:**
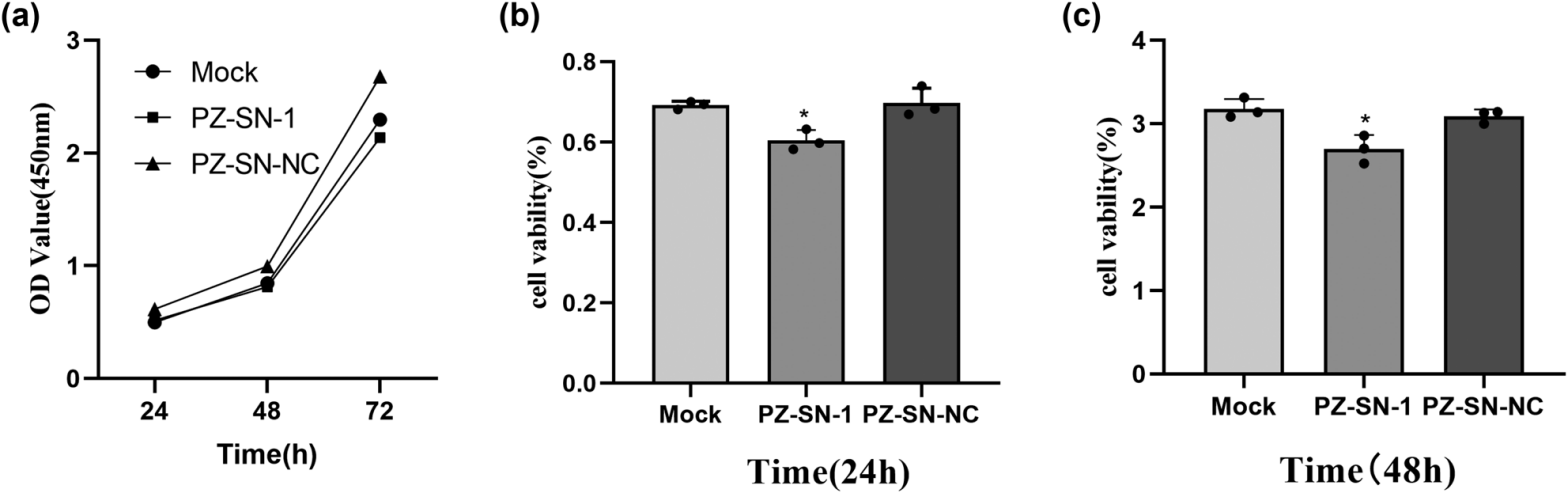
Influence of PZ knocking-down on cellular proliferation in A549 cells. (a) Cellular proliferation is examined with CCK-8 assay in the indicated A549 cells. The average proliferation rate is calculated from three independent assays in the cells for 24 h (b) and 48 h (c). * indicates the significant difference between the groups (*p* < 0.05).

The effect of the PZ protein on cellular proliferation was then examined in A549 cells using a CCK-8 kit. The results revealed that PZ knocking-down significantly prohibited the PZ-induced cellular proliferation compared with the mock transfection and siRNA-NC-transfected A549 cells within 24 h and 48 h of incubation (*p* < 0.05; Figure 4).

### 3.5 PZ-knockdown decreased the expression of Slug, Vimentin, and N-cadherin in A549 cells

**Figure 5:**
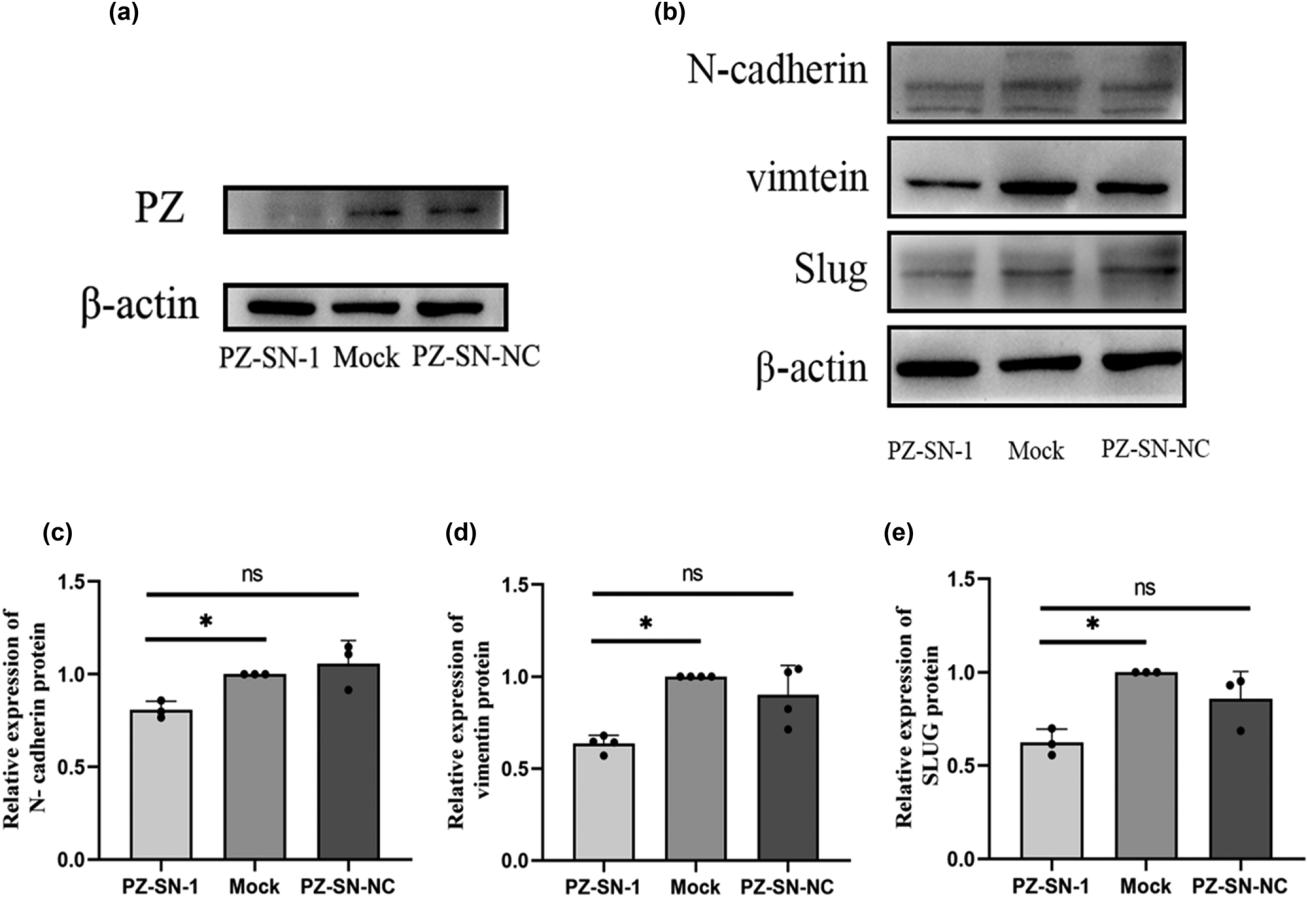
Effect of PZ knocking-down on epithelial–mesenchymal transition (EMT)-related proteins (Slug, Vimentin, and N-cadherin) expression in A549 cells. (a) Representative images of the Western blot shows the PZ (upper panel) expression in the PZ-siRNA-001 (left), mock (middle), and siRNA-NC (right) A549 cells. (b) The protein expression of N-cadherin (upper panel), Vimentin (second panel), and Slug (third panel) are examined by Western blot in the PZ-siRNA-1 (left), mock (middle), and siRNA-NC (right) cells. The average expression levels of N-cadherin (c), Vimentin (d), and Slug (e) in the corresponding cells are evaluated from three independent assays. * indicates the significant difference between the groups (*p* < 0.05).

To estimate the impact of the PZ on EMT, the EMT pathway-related proteins Slug, Vimentin, and N-cadherin were detected by Western blot in the PZ-siRNA-001 transfected A549 cells. Interestingly, the expression levels of those three proteins were significantly lower in the PZ-siRNA-transfected A549 cells compared to mock control cells and negative control cells (*p* < 0.05; Figure 5).

## 4 Discussion

In this study, the PZ protein was highly expressed in the NSCLC cell line (A549 cells). Knocking down PZ significantly inhibits the PZ-induced cellular proliferation, migration, invasion, and EMT-related protein expression in the A549 cells. These findings suggest that the pro-metastatic activity of the PZ may mediate through the EMT pathway.

PZ was expressed in three NSCLC cell lines (A549, H1299, H1975) in this study, which is in agreement with previous observations that PZ is expressed in various tumor tissues [16,17], including breast cancer [18,19], lung cancer [20], colon cancer, gastric cancer [21,22], and others [16,17]. Cellular proliferation, migration, and invasion are the primary cellular abilities of the metastasis of cancer cells. Reduction of cellular proliferation, cell migration, and cell invasion in the PZ-knocked-down A549 cells indicates that PZ might impact the metastasis of NSCLCs. Indeed, we have previously hypothesized that PZ plays a vital role in tumor growth, malignancy, and metastasis [24]. The data of this study further confirmed this opinion.

EMT is one of the main pathways involved in cancer metastasis. Slug [33], Vimentin [34], E-cadherin [42], and N-cadherin [39,40] are crucial proteins in the EMT process. The findings of Slug, Vimentin, and N-cadherin expression in A549 cells are entirely similar to the previous data in which Slug, Vimentin, and N-cadherin are expressed in multiple cancers, including breast [43], pancreatic [44], colon [45], and ovarian cancer [46,47]. The observation that PZ knocking-down significantly inhibited cell migration and invasion indicates that PZ is a pro-metastatic factor. It supports the previous findings that Slug, Vimentin, and N-cadherin play roles in enhancing cancer metastasis and suggests that the pro-metastasis effect of the PZ may involve EMT regulation in the A549 cells.

PZ protein and mRNA expression have been investigated in multiple tumor tissues. The results suggest that PZ might involve the metastatic regulation or anticoagulation mechanism in the neoplasm. However, there is still a gap in the details of the tumor metastatic regulation mechanism involved in PZ. Through knockdown gene technology, this study proved that the level of PZ expression in A549 cells directly affects the proliferation, migration, and tissue invasion of the tumor cells. It provides evidence at the cellular biological level for the speculation that PZ is involved in tumor metastasis. Further, we found that knocking-down PZ expression in A549 cells coincided with the downregulation of EMT essential proteins (Slug, Vimentin, and N-cadherin). On the basis of the aforementioned findings, we speculate that PZ protein controls the EMT process by regulating the expression of Slug, Vimentin, and N-cadherin, thus affecting the metastasis ability of tumor cells.

Although the present study first explored the pathological activity of PZ in lung adenocarcinoma and proved that PZ has a pro-metastatic ability and a possible mechanism, there were still a few deficiencies. First, this study did not conduct an in-depth analysis of the direct influence of PZ on the regulation of cellular proliferation, migration, and invasion by the gene or chemical interference of the essential EMT proteins (such as Slug, Vimentin, and N-cadherin). Second, only one silencing method was applied in this study. To silence the expression of PZ and examine the effects of PZ on migration and invasion, subsequent studies should use more methods such as shRNA and CRISPR, so that they will provide increasingly strong evidence to make a firm conclusion. Finally, the study did not perform animal experiments due to limited financial and technical resources.

## 5 Conclusions

PZ is expressed in lung adenocarcinoma cells. Knockdown of PZ decreases cell wound healing capacity, migration, invasion, and the protein levels of Slug, Vimentin, and N-cadherin in A549 cells, indicating that PZ modulates metastasis and invasion through the EMT pathway in lung cancer cells. These results provide new ideas for treating lung cancer, especially for the therapy of antitumor metastasis.
