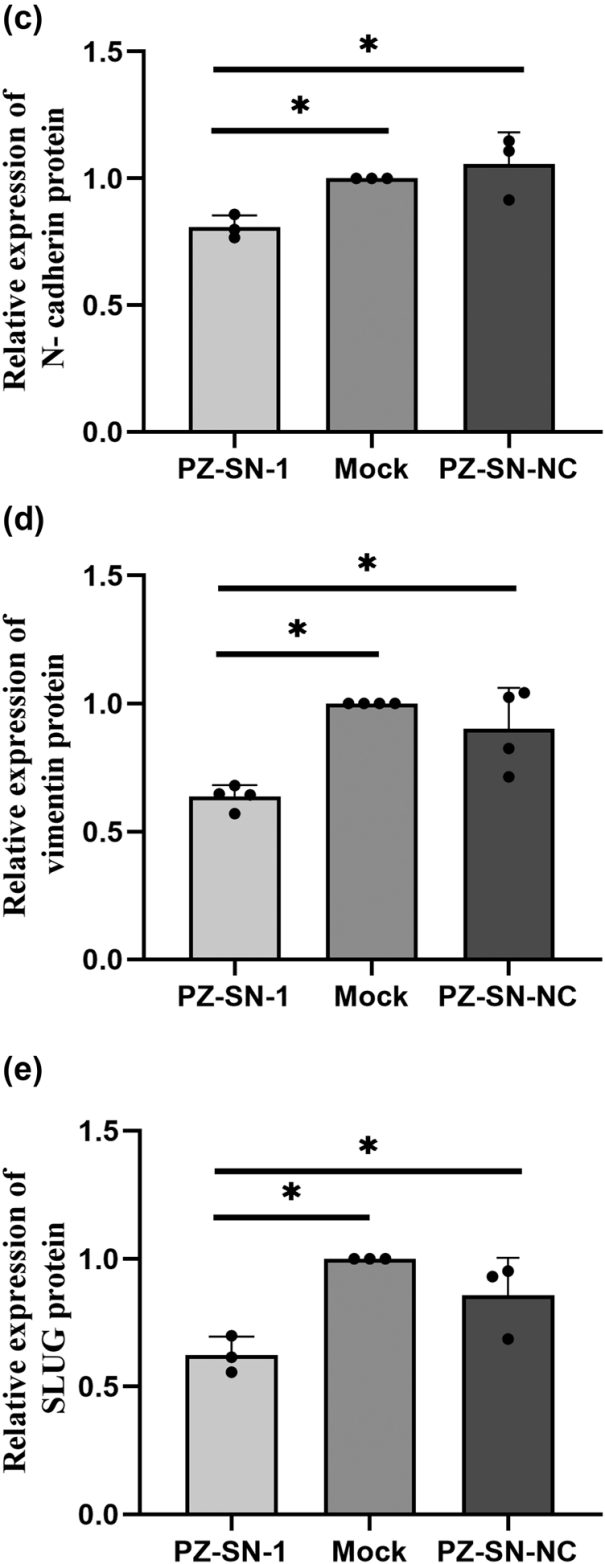# Erratum to “Protein Z modulates the metastasis of lung adenocarcinoma cells”

**DOI:** 10.1515/biol-2022-0715

**Published:** 2023-08-16

**Authors:** Jin Peng, Kai-Ying Yang, Huan Li, Shan-Shan Zheng, Xue-Yi Pan

**Affiliations:** Department of Hematology, The First Affiliated Hospital of Guangdong Pharmaceutical University, 19 Nong Lin Road, Yuexiu District, Guangzhou 510080 Guangdong, China

In the published article Peng J, Yang K, Li H, Zheng S, Pan X. Protein Z modulates the metastasis of lung adenocarcinoma cells. Open Life Sci. 2023;18(1):20220667, doi: 10.1515/biol-2022-0667, the authors found some marking errors in Figure 5. In the Figure 5c, Figure 5d and Figure 5e, “ns” should be changed to “*”. The figure legend is correct and does not need to be modified. The authors admit to the error and claim that this is an unintentional labeling error that has nothing to do with academic misconduct and does not influence the conclusion of the publication. The authors apologize to the editor, the staffs of the journal and the journal readers for the mistake and any inconvenience it caused.

**Figure 5:**